# False lumen/true lumen wall pressure ratio is increased in acute non-A non-B aortic dissection

**DOI:** 10.1093/icvts/ivac138

**Published:** 2022-05-13

**Authors:** Naoyuki Kimura, Masanori Nakamura, Reiya Takagi, Makiko Naka Mieno, Atsushi Yamaguchi, Martin Czerny, Friedhelm Beyersdorf, Fabian Alexander Kari, Bartosz Rylski

**Affiliations:** 1 Department of Cardiovascular Surgery, Saitama Medical Center, Jichi Medical University, Saitama, Japan; 2 Department of Electrical and Mechanical Engineering, Nagoya Institute of Technology, Nagoya, Japan; 3 Department of Medical Informatics, Center for Information, Jichi Medical University, Shimotsuke, Japan; 4 Department of Cardiovascular Surgery, University Heart Centre Freiburg, University of Freiburg, Freiburg, Germany

**Keywords:** Non-A non-B aortic dissection, Computational fluid dynamics, Wall pressure

## Abstract

**OBJECTIVES:**

We aimed to determine whether non-A non-B aortic dissection (AD) differs in morphologic and haemodynamic properties from type B AD.

**METHODS:**

We simulated and compared haemodynamics of patients with acute type B or acute non-A non-B AD by means of computational fluid dynamics. Wall pressure and wall shear stress (WSS) in both the true lumen (TL) and false lumen (FL) at early, mid- and late systole were evaluated. Morphology, WSS and the FL/TL wall pressure ratio were compared between groups.

**RESULTS:**

Nineteen patients (type B, *n* = 7; non-A non-B, *n* = 12) were included. The median age (51 [46, 67] vs 53 [50, 63] years; *P *=* *0.71) and a complicated course (14% vs 33%; *P *=* *0.6) did not differ between the type B group and the non-A non-B group. However, the median entry tear width was increased in the non-A non-B group (9.7 [7.3, 12.7] vs 16.3 [11.9, 24.9] mm; *P *=* *0.010). Streamlines showed, in patients with non-A non-B AD, blood from the TL flowed into the FL via the entry tear. Prevalence of a FL/TL wall pressure ratio >1.0 (type B versus non-A non-B) at early, mid- and late systole was 57% vs 83% (*P *=* *0.31), 43% vs 83% (*P *=* *0.13) and 57% vs 75% (*P *=* *0.62), respectively. WSS did not differ between the groups.

**CONCLUSIONS:**

The increased FL/TL wall pressure ratio observed during systole in non-A non-B AD may beget a complicated presentation.

**Figure:**
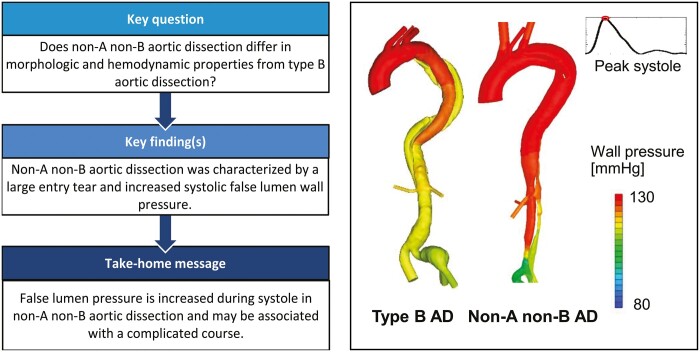

## INTRODUCTION

Acute non-A non-B aortic dissection (AD), a new concept of aortic pathology, is characterized morphologically by confinement of the dissection to the aortic arch or arch involvement either by the most proximal tear or by retrograde extension without extending to the ascending aorta [1, 2]. Acute non-A non-B AD is not a particularly rare condition, with a reported incidence of 11% among patients with acute AD [3]. An anomaly of the aortic arch is found more frequently in patients with non-A non-B AD than in those with other ADs [1, 3]. There is growing evidence that the clinical presentations of acute non-A non-B AD differ from those of acute type B AD without arch involvement; the clinical course is more likely to be complicated and requires intervention for patients with acute non-A non-B AD than for those with acute type B AD [2–4]. However, haemodynamic characteristics of acute non-A non-B AD remain to be elucidated.

Computational fluid dynamics (CFD) has been introduced into clinical practice to determine a patient’s haemodynamic variables, including flow streamlines, wall shear stress (WSS) and perpendicular pressure on the vessel wall. CFD has emerged as a useful tool for the assessment of haemodynamics in the area of AD, and flow rate/velocity, wall pressure and WSS are used in analysing haemodynamics in patients with AD [5–10]. However, analysis of the haemodynamic variables characteristic of acute non-A non-B AD has been reported only once, simply in a single case [11]. Herein, we describe results of a retrospective study in which we tested the feasibility of patient-specific CFD for investigation of haemodynamics in individuals with acute non-A non-B AD. We also describe the results of a comparative CFD-based study conducted to assess haemodynamics characterizing both acute non-A non-B AD and conventional acute type B AD.

## PATIENTS AND METHODS

### Ethics statement

This study was approved by the Institutional Review Board of University Heart Centre Freiburg (289/14) and Saitama Medical Center, Jichi Medical University (S20-11). The requirement for informed consent was waived.

### Patients and study design

Patients to be included in the study were selected through a review of the aortic databases maintained by 2 institutions: University Heart Centre Freiburg (Freiburg, Germany) and Saitama Medical Center, Jichi Medical University (Saitama, Japan). Those from the University Heart Centre Freiburg had been seen between April 2010 and March 2018, and those from Saitama Medical Center had been seen between November 2015 and February 2021. To ensure accurate CFD simulation, we applied the following exclusion criteria: (i) a totally or partially thrombosed false lumen (FL), (ii) 3- or 4-channeled AD, (iii) a highly tortuous aorta, (iv) chronic kidney disease requiring haemodialysis, (v) a calcified aorta, (vi) non-performance of contrast-enhanced computed tomography (CT) and (vii) low contrast enhancement within the FL, even if the FL was completely patent (reconstruction accuracy is not adequate for simulation in cases characterized by this feature).

**Figure 1:**
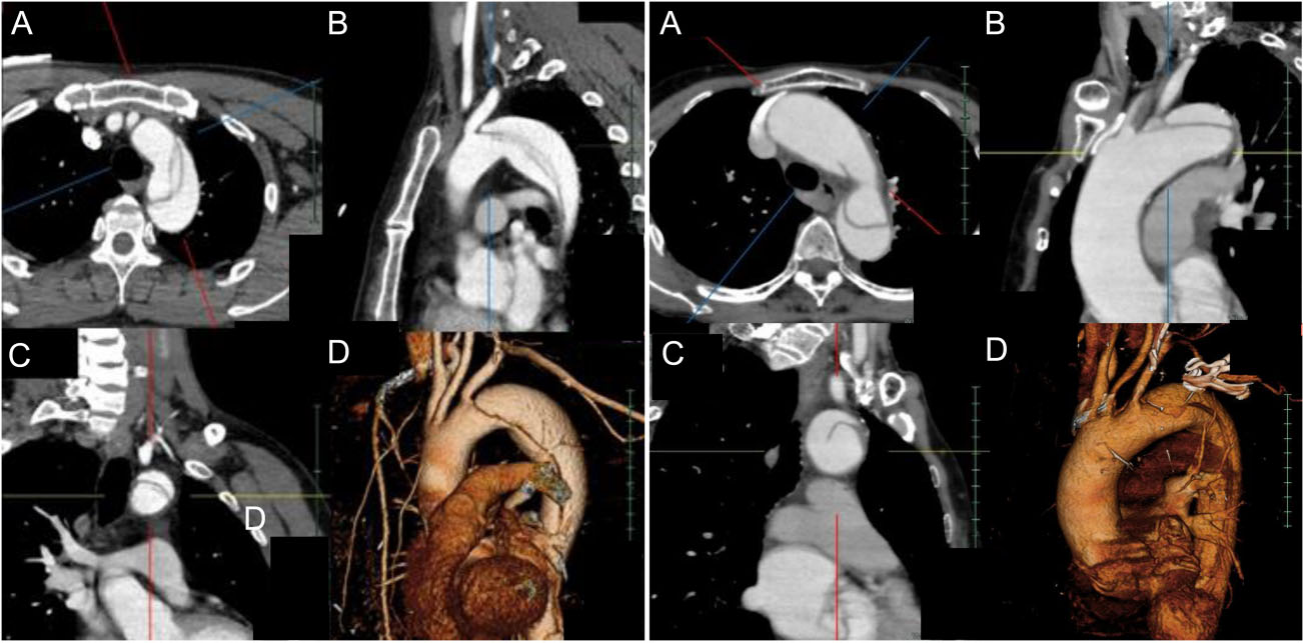
Multiplanar (**A**–**C**) and three-dimensional (**D**) reconstruction computed tomography angiography images obtained in a case of acute type B aortic dissection (left) and a case of acute non-A non-B arch-entry aortic dissection (right).

The type of AD, i.e. type B or non-A non-B AD, was diagnosed by means of multiplanar CT angiography (Fig. 1). Patient characteristics (age, sex, Marfan syndrome, hypertension, clinical presentation and treatment), morphologic characteristics (arch configuration, distal extent of dissection and location and size of the entry tear) and treatment outcomes (in-hospital survival) as well as haemodynamic variables (wall pressure and WSS) were compared between the acute type B AD group and the acute non-A non-B AD group.

### Imaging analysis and computer simulation of blood flow

All patients included in the study had undergone contrast-enhanced CT angiography on admission at either of the 2 hospitals. The imaging had been performed with a SOMATOM Definition Flash scanner (Siemens Healthcare, Munich, Germany). Scans were obtained with a slice thickness of 1 mm. Analysis was performed with Impax EE (Agfa HealthCare N.V., Morstel, Belgium) at University Heart Centre Freiburg or Synapse Vincent (Fujifilm Inc., Tokyo, Japan) at Saitama Medical Center, Jichi Medical University. The CT scan covered the entire aorta including the 3 main branches arising from the aortic arch and the origin of common iliac arteries. Width of the entry tear and distance between the entry tear and the left subclavian artery were measured on multiplanar reconstruction images, always in the plane perpendicular to the manually corrected local aortic centre line drawn from the aortic arch to the aortic bifurcation [12].

The three-dimensional (3D) geometry of the aorta was reconstructed from preoperative CT angiography data, which were approved by cardiac surgeons. The resultant CT angiograms had a spatial resolution of 0.7812 mm × 0.7812 mm × 1 mm. The intraluminal region of interest was reconstructed with open-source imaging software, 3D Slicer (ver. 4.10.2). Although the abdominal arteries were included when they were clearly visible, we confirmed that their effects on haemodynamics were not large (Supplementary Material, Fig. S1 and Supplementary Material, Table S1). The surface was smoothed with a commercially available digital sculpting program, 3DCoat (ver. 4.1.17d, Pilgway, Kiev, Ukraine). In mesh generation, 5 layers of prism mesh were created on the surface, and the remaining region was filled with tetrahedral mesh. The total number of mesh elements was approximately 0.5–1 million. Note that mesh independence was confirmed for this number of mesh elements (Supplementary Material, Fig. S2).

**Figure 2:**
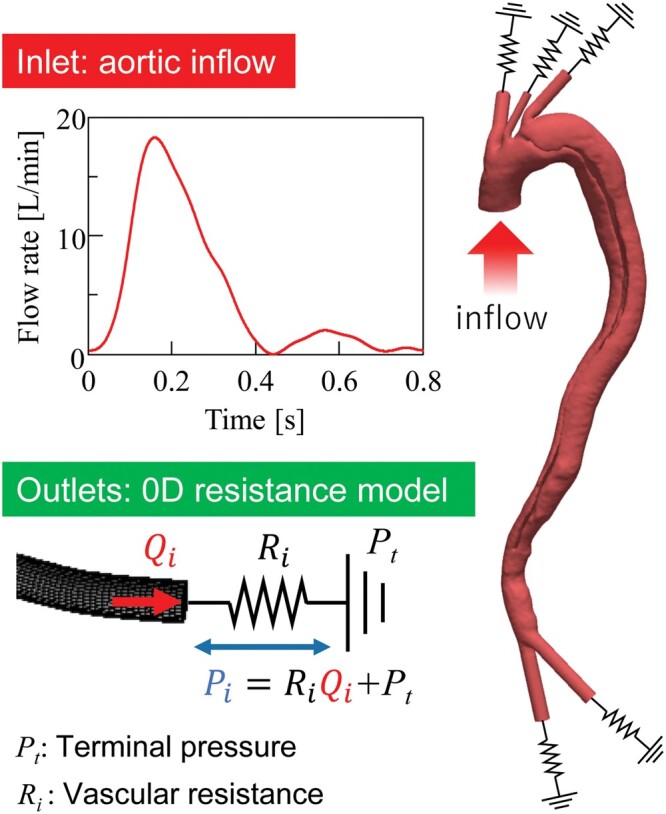
Procedural steps for patient-specific computational fluid dynamics simulation of blood flow.

Haemodynamics were simulated with a commercially available CFD program (scFLOW ver. 2020, Cradle, Japan) that adopts a finite volume method for the formulation. Blood was assumed to be an incompressible Newtonian fluid, with non-Newtonian behaviour not being particularly apparent in larger arteries. Density and dynamic viscosity were set to 1.05 × 10^3^ kg/m^3^ and 4.0 × 10^−3^ Pa/s, respectively. The Navier–Stokes equations of blood flow in a 3D domain were numerically coupled to peripheral blood flow models in a 0D domain (Fig. 2). The peripheral blood flow models were created based on the hydraulic-electric analogue where vascular resistance, a pressure difference, and mass flow are represented by electrical resistance, a potential difference, and electrical current, respectively. Thus, we used the resistance-type outlet boundary conditions for 3D flow simulation. The boundary condition at the downstream end of the 0D model was the capillary pressure, *P_t_* = 30 mmHg [13]. Vascular resistance *R* was determined by means of a structured tree model [13–15]. Note that the total resistance *R* was tuned in some cases in which the pressure obtained by the simulation was unphysiologically high. It was changed such that the spatiotemporally maximum pressure fell within physiological range (100–130 mmHg). At the aortic root, a physiological flow rate, created with some modifications of the measured data, was assigned as an inlet boundary condition. The wall was assumed to be rigid, and a no-slip boundary condition was applied.

Wall pressure perpendicular to the aortic wall and WSS were both evaluated at early systole, mid-systole and late systole, and these values were averaged over the surface area of the true lumen (TL) or FL in the descending thoracic aorta (Supplementary Material, Fig. S3).

Other information pertaining to computer simulation of blood flow is shown in the Supplementary Material.

### Statistical analysis

Continuous variables are reported as median (25th, 25th percentile) values. Categorical variables are reported as *n* (%). Between-group differences in continuous data were analysed by Mann–Whitney rank-sum test. Differences in categorical variables were analysed by Fisher’s exact test. All statistical analyses were performed with IBM SPSS Statistics 26.0 for Windows (IBM Corp., Armonk, NY, USA), and *P *<* *0.05 was considered significant.

## RESULTS

### Patient characteristics and treatment outcomes

A total of 232 patients with acute type B AD and 56 patients with acute non-A non-B AD had been admitted to the 2 hospitals. From among these patients, we selected 19 for CFD simulation analysis. These were patients with a completely patent FL and for whom CT images were of sufficient quality for CFD analysis. The 19 patients were of 2 groups: those with acute type B AD (AD without arch involvement, *n* = 7) and those acute non-A non-B AD group (AD that involved the aortic arch but did not extend into the ascending aorta, *n* = 12). Patient characteristics and treatment outcomes are shown per group in Table 1. Neither age (type B group, 51 [46, 67] years; non-A non-B group, 53 [50, 63] years; *P *=* *0.71) nor the prevalence of male sex [100% (7/7) vs 58% (7/12); *P *=* *0.11] differed significantly between the groups. Although the prevalence of aortic rupture and that of organ malperfusion were increased in the non-A non-B group, the differences were not significant. One patient with type B AD and 4 patients with non-A non-B AD underwent urgent surgical intervention, i.e. thoracic endovascular aortic repair (TEVAR) (*n* = 2) or aortic arch repair (*n* = 3). The remaining 14 patients, including 6 with type B AD and 8 with non-A non-B AD, were treated conservatively. One patient with non-A non-B AD, a 63-year-old woman whose case was complicated by cerebral malperfusion, died of multiorgan failure and cerebral damage after TEVAR. The entry tear was located in the aortic arch in 9 patients with non-A non-B AD and in the descending thoracic aorta in the remaining 3. Width of the entry tear was increased in the non-A non-B group (9.7 [7.3, 12.7] mm vs 16.3 [11.9, 24.9] mm; *P *=* *0.010). We observed that the distance from the entry tear to the left subclavian artery tended to be relatively short in the non-A non-B group (11.8 [4.3, 23.9] mm vs 4.4 [0.3, 15.3] mm, *P *=* *0.29).

**Table 1:** Patient characteristics and treatment outcomes, per type of aortic dissection

	Type B	Non-A non-B	*P*-Value
	(*n* = 7)	(*n* = 12)	
Age (years)	51 [46, 67]	53 [50, 63]	0.71
Male sex	100 (7)	58 (7)	0.11
Marfan syndrome	14 (1)	8 (1)	1.0
Bicuspid aortic valve	0 (0)	8 (1)	1.0
Hypertension	71 (5)	83 (10)	0.60
Uncomplicated	86 (6)	67 (8)	0.60
Aortic rupture	0 (0)	17 (2)	0.51
Malperfusion			
Cerebral	0 ( (0)	8 (1)	1.0
Kidney	0 (0)	8 (1)	1.0
Lower limb	14 (1)	8 (1)	1.0
Treatment			
Conservative	86 (6)	67 (8)	0.60
Thoracic endovascular aortic repair	14 (1)	8 (1)	1.0
Aortic arch repair without FET	0 (0)	17 (2)	0.51
Aortic arch repair with FET	0 (0)	0 (0)	1.0
In-hospital mortality	0 (0/7)	8 (1/12)	1.0
Conservative treatment	0 (0/6)	0 (0/8)	1.0
Thoracic endovascular aortic repair	0 (0/1)	100 (1/1)	1.0
Aortic arch repair	NA	0 (0/3)	NA
Aortic arch configuration			
Common orifice of the brachiocephalic trunk and the left common carotid artery	29 (2)	17 (2)	0.60
Arch origin of the left vertebral artery	0 (0)	0 (0)	1.0
Distal extension of dissection			
Limited to descending thoracic aorta	0 (0)	8 (1)	1.0
Abdominal aorta	29 (2)	58 (7)	0.35
Iliac artery	71 (5)	42 (4)	0.17
Entry location			
Arch convexity	0 (0)	67 (8)	0.013
Arch concavity	0 (0)	8 (1)	1.0
Descending thoracic aorta	100 (7)	25 (3)	0.003
Width of entry tear (mm)	9.7 [7.3, 12.7]	16.3 [11.9, 24.9]	0.010
Entry tear distance to LSA (mm)	11.8 [4.3, 23.9]	4.4 [0.3, 15.3]	0.29

Data are shown as median [25th, 75th percentile] values or % (number) of patients.

FET: frozen elephant trunk; LSA: left subclavian artery; NA: not available.

### Patient-specific simulation of haemodynamics

**Figure 3:**
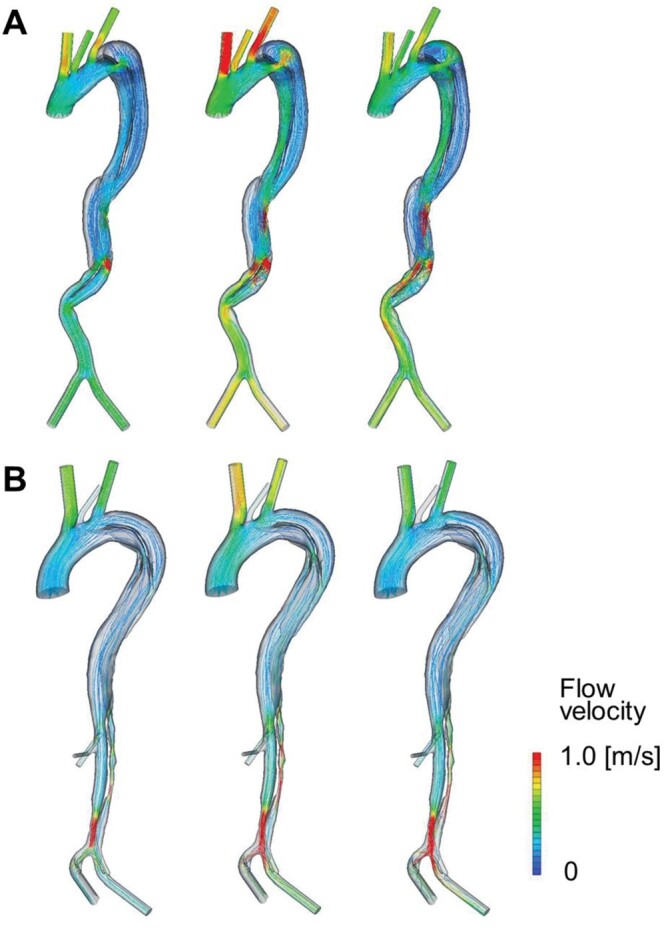
Streamline analysis of representative cases. (**A**) Acute type B aortic dissection and (**B**) acute non-A non-B aortic dissection.

Streamline patterns in representative cases of acute type B AD and acute non-A non-B AD are shown in Fig. 3 [Video 1 (type B) and Video 2 (non-A non-B)]. The streamlines were calculated by computing and plotting the trajectories of fluid elements within the dissected aorta. In the case of acute type B AD, blood entered the FL from the TL via an entry tear in the proximal descending thoracic aorta at early systole, and the flow began to spiral in the FL at mid-systole. Blood flow in the TL was laminar, and flow velocity in the TL reached its peak at a point of narrowing at mid-systole (Fig. 3A). In the case of acute non-A non-B AD, blood flowed, at early systole, from the TL to FL via an entry tear located in the aortic arch (zone 2). Blood flow was mostly laminar in both the TL and FL of the descending thoracic aorta during the entire systolic phase (Fig. 3B).

**Figure 4:**
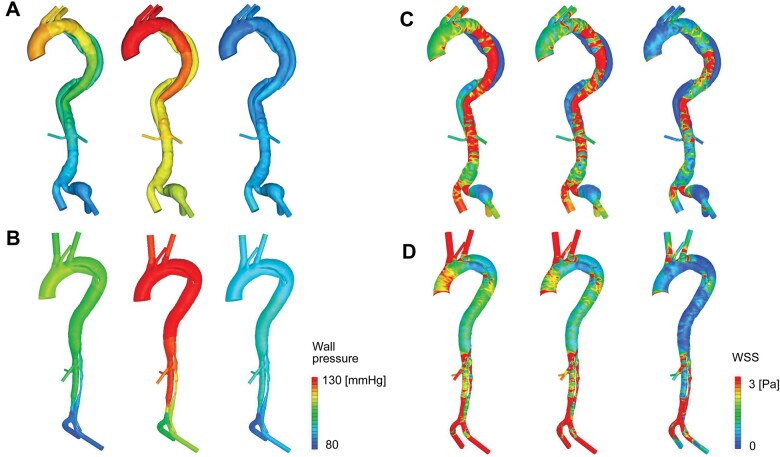
Representative images of aortic wall pressure and wall shear stress during systole, i.e. at early (left), mid- (middle), and late systole (right) for each analysis. Imaging data from a case of acute type B aortic dissection are shown in (**A**) (wall pressure) and (**C**) (wall shear stress), and imaging data from a case of acute non-A non-B aortic dissection are shown in (**B**) (wall pressure) and (**D**) (wall shear stress).

**Figure 5:**
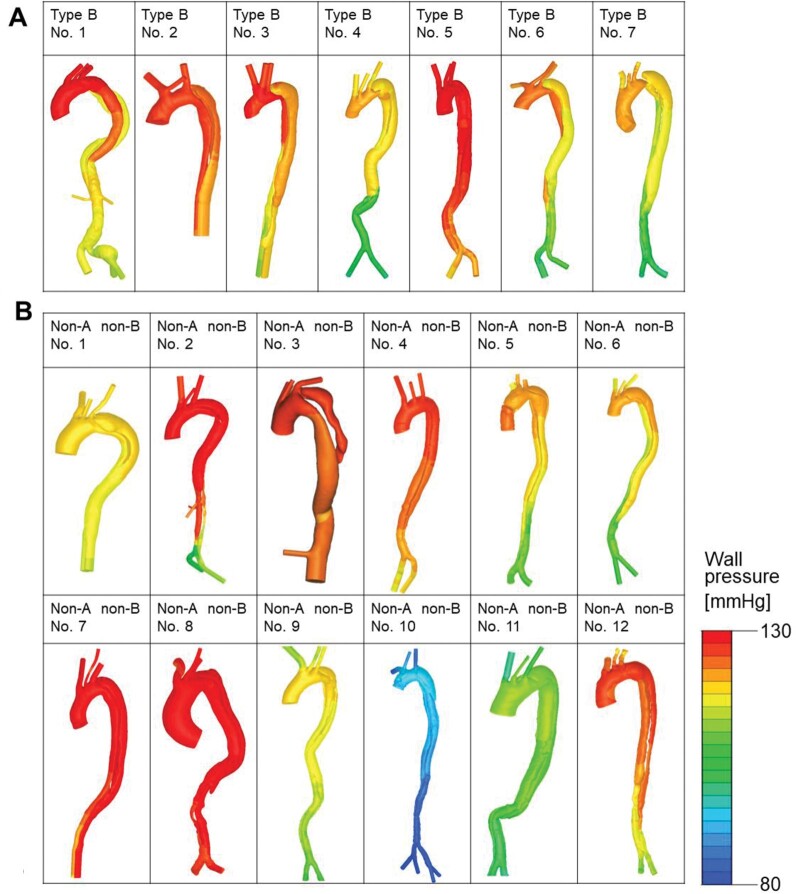
Aortic wall pressure at mid-systole. (**A**) Seven patients with acute type B aortic dissection. (**B**) Twelve patients with acute non-A non-B aortic dissection. Patients 7, 10 and 12 had an entry tear in the proximal descending thoracic aorta. The remaining 9 patients had an entry tear in the arch.

**Figure 6:**
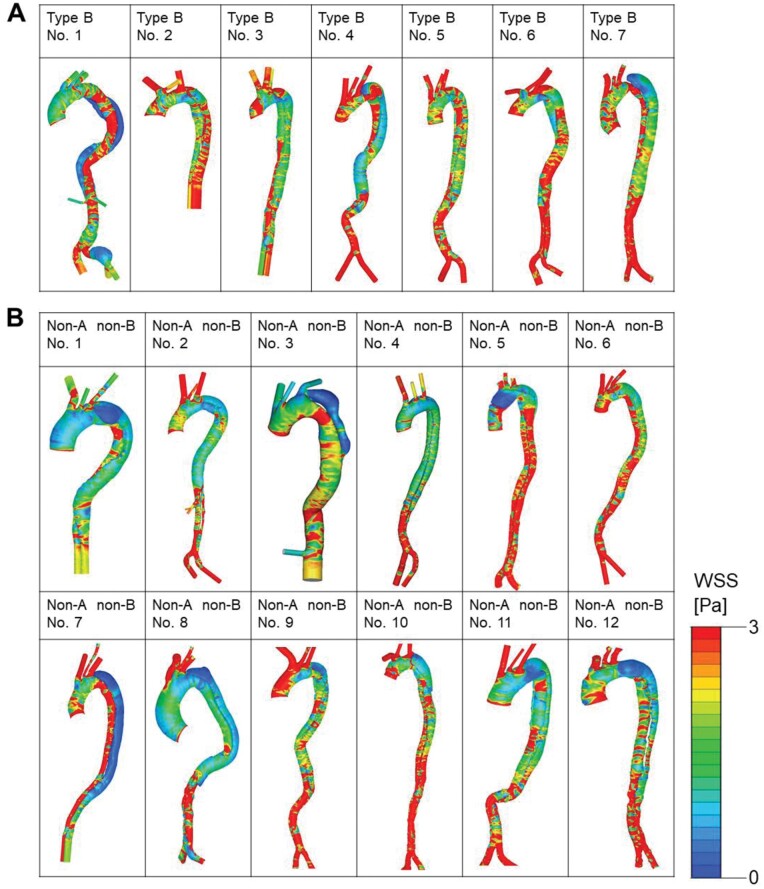
Wall shear stress at mid-systole. (**A**) Seven patients with acute type B aortic dissection. (**B**) Twelve patients with acute non-A non-B aortic dissection. Patients 7, 10, and 12 had an entry tear in the proximal descending thoracic aorta. The remaining 9 patients had an entry tear in the arch.

Representative images of aortic wall pressure and spatial distribution of WSS in cases of acute type B AD and acute non-A non-B AD are shown in Fig. 4. In the case of type B AD, aortic wall pressure was increased in the TL in comparison to that in the FL at early and mid-systole, after which, at late systole, the pressure dropped to a similar level in both lumens (Fig. 4A). Variations in pressure at the aortic inlet along with flowrate curves are shown in Supplementary Material, Fig. S4. In the case of non-A non-B AD, wall pressure was similar in both lumens throughout the systolic phase, although the degree of pressure differed at the different time points (Fig. 4B). WSS distribution was predominantly increased in the TL in the case of type B AD (Fig. 4C), and distribution of WSS was similar in both lumens in the case of non-A non-B AD (Fig. 4D). Contour plots of aortic wall pressure at mid-systole (anteroposterior view) are shown for all patients in Fig. 5. Wall pressure in the TL was higher than that in the FL in 4 of the 7 patients with type B AD (patients 1, 2, 3, and 6) (Fig. 5A). No increase in wall pressure in the TL relative to that in the FL was seen in any patient in the non-A non-B group. Two patients (patients 6 and 12) in this group showed slightly increased wall pressure in the FL. The remaining 10 patients showed similar wall pressure in both lumens (Fig. 5B). Contour plots of WSS distribution at mid-systole (anteroposterior view) are shown for all patients in Fig. 6. Seven patients with type B AD (Fig. 6A) and 12 patients with non-A non-B AD (Fig. 6B) showed heterogenous and complex patterns of WSS distribution in the descending thoracic aorta and in the abdominal aorta at mid-systole.

**Figure 7:**
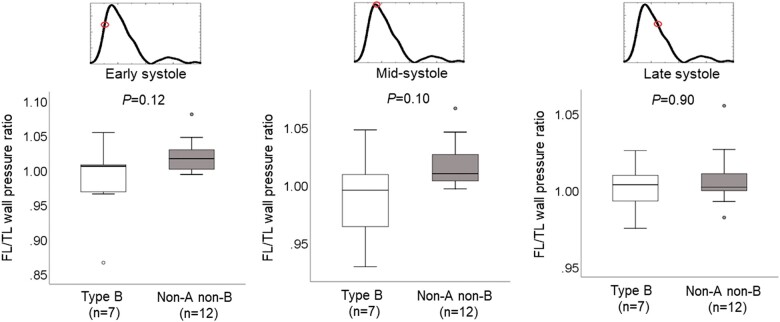
Box and whisker plots of false lumen/true lumen wall pressure ratio in patients with acute type B aortic dissection and acute non-A non-B aortic dissection. Wall pressure was measured in the false lumen and true lumen at early, mid-, and late systole. Wall pressure was subsequently averaged over the surface area. Median (horizontal line dissecting the box), interquartile range (length of the box), and minimum and maximum (whisker ends) values are shown. Outliers are shown as circles. *P* values were obtained by Mann–Whitney *U*-test.

We examined haemodynamic variables quantitatively. First, we compared haemodynamic variables between the TL and FL in each study group. As shown in Supplementary Material, Table S2, wall pressures did not differ significantly in either group. However, FL WSS was decreased in comparison to TL WSS in the non-A non-B AD group. Next, we compared the FL/TL wall pressure ratio at 3 time points during systole between the type B AD group and the non-A non-B AD group (Fig. 7). The FL/TL wall pressure ratio at early systole, mid-systole, and late systole for type B vs non-A non-B AD was 1.01 [0.97, 1.01] vs 1.02 [1.0, 1.03] (*P *=* *0.12), 0.99 [0.96, 1.01] vs 1.01 [1.0, 1.03] (*P *=* *0.10) and 1.0 [0.99, 1.01] vs 1.0 [1.0, 1.01] (*P *=* *0.90), respectively. Prevalence of an FL/TL wall pressure ratio >1.0 (type B vs non-A non-B) at early, mid- and late systole was 57% vs 83% (*P *=* *0.31), 43% vs 83% (*P *=* *0.13) and 57% vs 75% (*P *=* *0.62), respectively. Subsequently, we compared TL and FL WSS at 3 time points during systole. TL WSS was higher than FL WSS in both groups at all 3 time points. TL and FL WSS did not differ significantly between the type B group and the non-A non-B group (Supplementary Material, Fig. S5).

Finally, we compared the FL/TL pressure ratio between patients with a small entry tear (entry tear width <10 mm, *n* = 9) and those with a large entry tear (entry tear width >10 mm, *n* = 10), regardless of the type of AD. Case characteristics and FL/TL ratio per size of the entry tear are shown in Supplementary Material, Table S3. Patients with a large entry tear were somewhat more likely to have a complicated course and increased FL/TL ratio during systole.

## DISCUSSION

The term ‘non-A non-B’ AD was initially proposed by von Segesser *et al.* [16] in 1994 to describe descending thoracic AD with extension of the dissection into the aortic arch. According to the 2019 expert consensus document of the European Association for Cardio-Thoracic Surgery and the European Society for Vascular Surgery, non-A non-B AD was defined as arch involvement either by the most proximal tear or by retrograde extension but without dissection involving the ascending aorta [1]. In this study, we investigated, by means of CFD, haemodynamics of acute non-A non-B AD in comparison to haemodynamics of acute type B AD. We believe our study to be the first blood flow analysis-based investigation of the haemodynamic characteristics of acute non-A non-B AD in a series of patients, and through our study, we identified a blood flow pattern specific to non-A non-B AD. We also showed increased FL wall pressure during systole in patients with non-A non-B AD.

Controversy remains regarding the effect of arch involvement on outcomes of acute AD. Some investigators have shown outcomes to be comparable between acute type B AD with and without arch involvement [17, 18]. Nauta *et al.* [18] reported that 16.5% (67/404) of patients with acute type B AD had retrograde arch dissection, and clinical presentation (organ malperfusion and rupture), treatment applied, and in-hospital mortality were similar between acute type B AD with and without arch involvement. In contrast, recent studies have shown patients with non-A non-B AD are more likely than those with type B AD to have a complicated clinical presentation. We reported previously that emergency open surgical repair or TEVAR was necessary due to malperfusion or rupture in 29% of cases of descending-entry type acute non-A non-B AD and 36% of cases of arch-entry type acute non-A non-B AD [3]. A recent meta-analysis also showed 30-day mortality of patients with acute non-A non-B AD treated with conservative therapy to be 14% (7/50) [2], greater than that of patients with acute type B dissection [19]. Hybrid aortic repair has recently been reported to yield favourable outcomes in cases of non-A non-B AD [20]. We should perform the most suitable treatment promptly based on clinical presentation of the non-A non-B AD.

CFD is a powerful investigative tool applicable to cardiovascular research. Combined use of CFD and imaging modalities allows for patient-specific simulation of haemodynamics. CFD has been widely used in the field of AD research [5–9, 11]. Several haemodynamic conditions, including high WSS, difference in wall pressure between the TL and FL, and a high FL flow rate, have been implicated in the pathogenesis of organ malperfusion, rupture, and late aneurysm formation in type B AD [5, 6]. Of these conditions, a large difference in wall pressure between lumens is considered to promote TL compression in acute type B AD [5, 7, 9].

We showed that the prevalence of FL/TL wall pressure >1.0 was increased at all 3 time points during systole in our non-A non-B AD group. This finding suggested that the increased FL pressure causes TL compression, in turn leading to organ malperfusion and/or aortic rupture. Several groups of investigators have, using CFD, examined influence of the entry tear on the pathogenesis of type B AD [7–9]. D’Ancona *et al.* [8], from a CFD-based study of 24 patients with type B AD, reported a larger entry tear resulted in greater FL flow. Rinaudo *et al.* [7] studied 25 patients with type B AD, then showed that FL flow was significantly greater in patients with an entry tear >10 mm (*n* = 17) than in those with an entry tear <10 mm (*n* = 8). Previous non-CFD morphologic studies revealed that a large entry tear [21, 22] and an entry tear close to the left subclavian artery [22, 23] increase the risk of adverse events in cases of type B AD. In the study reported herein, 67% (8/12) of our patients with non-A non-B AD had a large entry tear (>10 mm); thus, the prevalence was higher than that among patients with type B AD (29%, 2/7). Furthermore, in our non-A non-B AD group, the median distance between the entry tear and left subclavian artery was 4.4 mm. We hypothesize that both a large entry tear and the tear being in close proximity to the left subclavian artery are associated with elevation of FL wall pressure in acute non-A non-B AD.

Few studies have involved investigation of the morphologic characteristic of non-A non-B AD. We reported previously the entry tear was located in the convexity of the arch in 21 of 22 patients with arch-entry non-A non-B AD [3]. Similarly, in the study reported herein, we found the entry tear to be located in the convexity of the arch in 87% (7/8) of patients with arch-entry non-A non-B AD. We also reported previously the frequency of an arch anomaly is increased among patients with non-A non-B AD [3]. However, the frequency of an arch anomaly did not differ between our study groups.

### Limitations

Our investigation had several limitations. First and foremost was the small sample size, and the small number of patients with non-A non-B AD prevented quantitative comparison of haemodynamic variables between arch-entry and descending-entry non-A non-B AD. Second, we selected the study patients on the basis of specific criteria—i.e. particular anatomical features and suitability of their imaging data—possibly introducing patient selection bias. Third, compliance of the aortic wall was not considered in our CFD model. The assumed rigidity could have resulted in overestimation or underestimation of the aortic wall pressure and WSS. In fact, in the present study, the pressure decreased faster in the transition from systole to diastole, and the pressure declined to approximately 30 mmHg during diastole due to a lack of aortic compliance. Such pressure variations are unphysiological. A recent *ex vivo* study in porcine aorta showed an aortic wall with reduced elasticity results in an increased FL diameter [24]. However, inclusion of elasticity as a study variable requires a fluid–structure interaction simulation model. The material properties necessary for the structure simulation are difficult to obtain non-invasively. Determining the material properties of the intimal flap would be difficult in cases of AD; because of their locations, they would be heterogeneous. Furthermore, residual stresses within the aortic wall are unknown, making the solid analysis more challenging. Yet, fluid–structure interaction simulations will yield a more accurate description than the assumption of rigid walls, thereby providing for quantitative discussions. Although the numerical complexity increases significantly with such studies, and they may be subject to measurement uncertainties, we believe such simulations should be included in future studies.

## CONCLUSION

Patient-specific CFD-based assessment of haemodynamics is clinically feasible in cases of acute non-A non-B AD. We found that entry tear width was increased in our non-A non-B AD group patients. We also found that the prevalence of FL/TL wall pressure >1.0 during systole was increased in this group, and this may be associated with unstable conditions in patients with non-A non-B AD. Studies that include large numbers of patients are needed to validate our findings.

## SUPPLEMENTARY MATERIAL

Supplementary material is available at *ICVTS* online.

## Funding

This work was supported by a Grant-in-Aid for Scientific Research C (# 21K08828 to Naoyuki Kimura) from the Ministry of Education, Culture, Sports, Science and Technology, Japan.

**Conflict of interest:** none declared.

## Data Availability Statement

All relevant data are within the manuscript and its Supporting Information files.

## Author contributions

**Naoyuki Kimura:** Conceptualization; Data curation; Funding acquisition; Investigation; Methodology; Resources; Validation; Writing—original draft; Writing—review & editing. **Masanori Nakamura:** Conceptualization; Data curation; Investigation; Methodology; Software; Writing—original draft. **Reiya Takagi:** Investigation; Software. **Makiko Naka Mieno:** Data curation; Supervision. **Atsushi Yamaguchi:** Supervision. **Martin Czerny:** Supervision. **Friedhelm Beyersdorf:** Supervision. **Fabian Alexander Kari:** Supervision. **Bartosz Rylski:** Conceptualization; Validation; Visualization.

## Reviewer information

Interactive CardioVascular and Thoracic Surgery thanks the anonymous reviewer(s) for their contribution to the peer review process of this article.

## Supplementary Material

ivac138_Supplementary_Data

## Notes

Presented at the 35th Annual Meeting of the European Association for Cardio-Thoracic Surgery, Barcelona, Spain, 13–16 October 2021.

## ABBREVIATIONS

AD: Aortic dissection
CFD: Computational fluid dynamics
CT: Computed tomography
3D: Three-dimensional
FL: False lumen
TEVAR: Thoracic endovascular aortic repair
TL: True lumen
WSS: Wall shear stress
