# Supplementary material for: False lumen/true lumen wall pressure ratio is increased in acute non-A non-B aortic dissection
Source: Interact Cardiovasc Thorac Surg. 2022 May 13;35(3):ivac138. doi: 10.1093/icvts/ivac138 (PMC9486891; doi:10.1093/icvts/ivac138)
Supplement: ivac138_Supplementary_Data [file ivac138_supplementary_data.zip › Supplementary Files.docx]

**Supplementary Data**

**METHODS**

**Computer simulation of blood flow**

With the structured tree model used, a parent blood vessel symmetrically branches into two daughter vessels whose diameter and length are determined in reference to the diameter of the parent blood vessel. Branching starts from each outlet of the 3D aortic model and continues recursively until the diameter of the smallest daughter branch reaches that of the arterioles (0.015 mm). The process yields a fractal vascular network model. Resistance of each daughter branch is determined by application of the Hagen-Poiseuille equation, and total resistance *R* of the entire tree was obtained.

The actual 3D flow simulation was begun with a capillary outlet pressure boundary condition of *P_t_* = 30 mmHg. The flowrate obtained at each outlet by the 3D flow simulation was transferred to the 0D model to determine the outlet blood pressure of the 3D model for the next time step. The 3D flow simulation was then implemented with the updated pressure boundary conditions, and the obtained flowrates were again transferred to the 0D model to further update the pressure outlet boundary conditions for the 3D simulation. This process was progressively repeated to simulate blood flow in the aorta.

**Figure S1 Table S1**

|  | *P_TL_* [mmHg] | *P_FL_* [mmHg] | *P_FL_/P_TL_* |
| --- | --- | --- | --- |
| With abdominal arteries | 127.5 | 128.0 | 1.004 |
| W/O abdominal arteries | 132.2 | 133.0 | 1.006 |

**
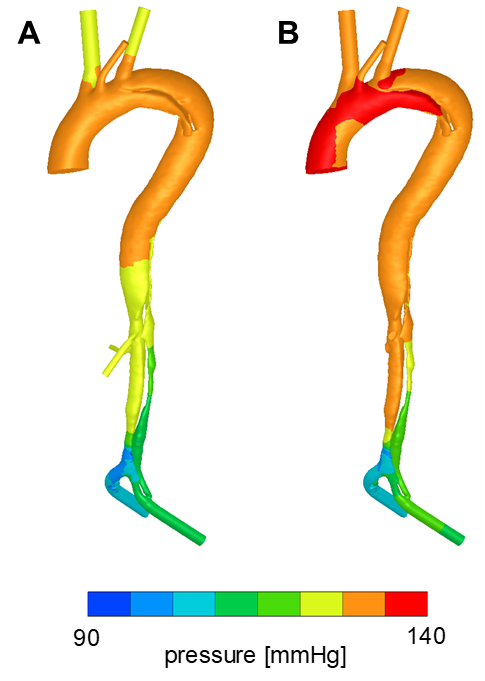
**

**Table S1**: Pressure in the true lumen and false lumen in the geometry models with and without the abdominal arteries. P*_TL_*: Pressure in the true lumen; P*_FL_*: Pressure in the false lumen.

**Figure S1**: Contour plots of the pressure at peak systole in the geometry model (**A**) with the abdominal arteries and in that (**B**) without the abdominal arteries.

**Figure S2**

**
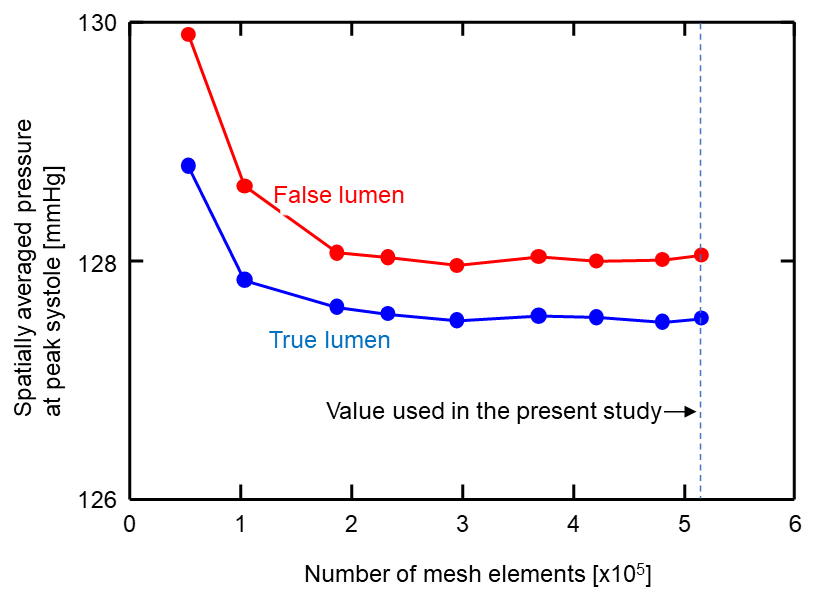
**

**Figure S2**: Results of mesh independence study.

**Figure S3**


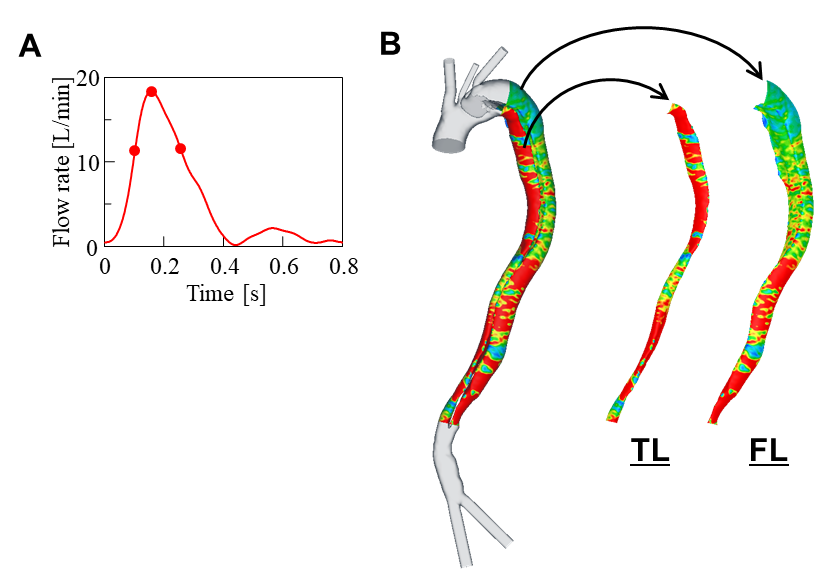


**Figure S3**: Analysis of CFD parameters per TL and FL. (**A**) Wall pressure perpendicular to the aortic wall and WSS were assessed in both the TL and FL during the systolic phase-- at early systole, mid-systole, and late systole. (**B**) WSS was averaged over the surface area of the TL or FL at each time point. CFD: computational fluid dynamics; WSS: wall shear stress; TL: true lumen; false lumen.

**Figure S4**


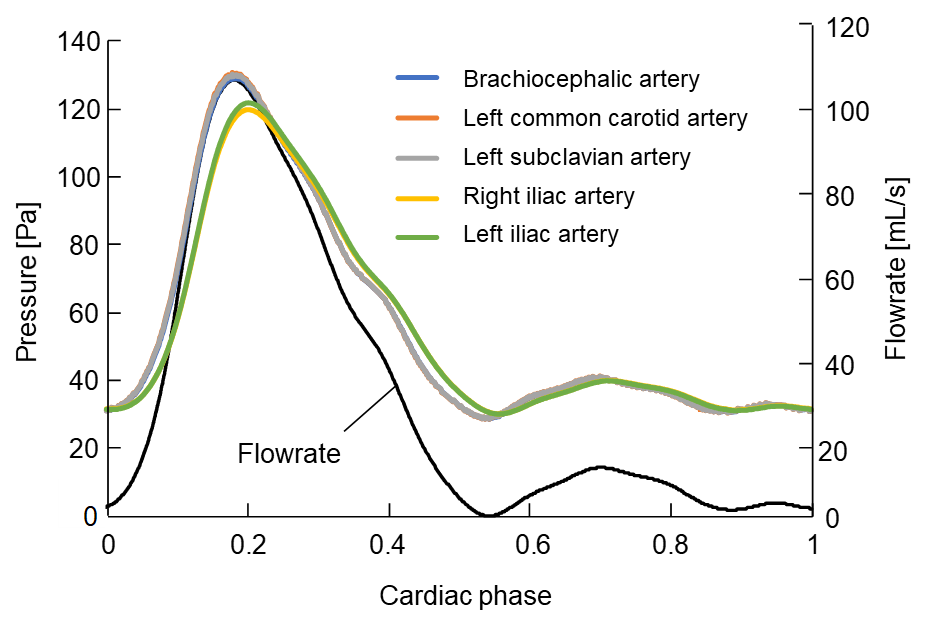


**Figure S4**: Pressure in relation to flowrates at the aortic inlet.

**Figure S5**

**
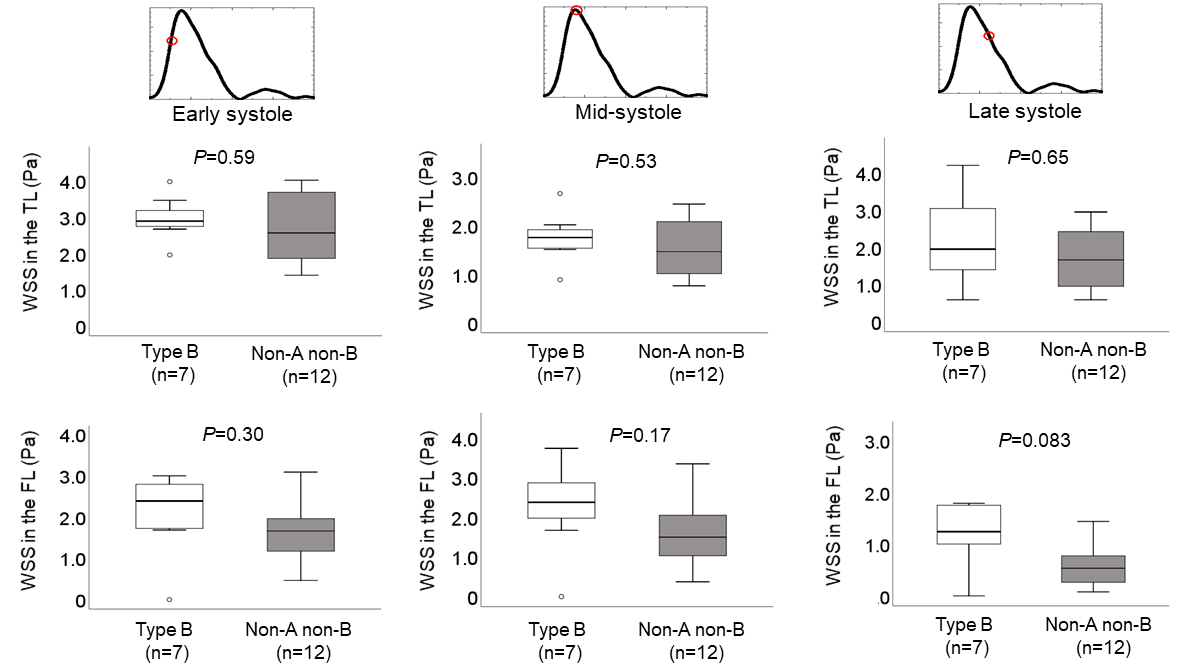
**

**Figure S5**: Box and whisker plots of WSS within the TL and FL in patients with acute type B AD and those with acute non-A non-B AD. WSS was measured in both lumens at early, mid-, and late systole. WSS was subsequently averaged over the surface area. Median (horizontal line dissecting the box), interquartile range (length of the box), and minimum and maximum (whisker ends) values are shown. Outliers are shown as circles. *P* values were obtained by Mann-Whitney U-test. AD: aortic dissection; FL: false lumen; TL: true lumen.

**Table S2:** Wall pressure and WSS in the TL and FL, shown for each group

|  | TL | FL | *P* value |
| --- | --- | --- | --- |
| Acute type B AD group (n=7) |  |  |  |
| Wall pressure |  |  |  |
| Early systole (mmHg) | 138 [131, 144] | 134 [132, 139] | 0.81 |
| Mid-systole (mmHg) | 161 [153, 168] | 156 [154, 164] | 0.71 |
| Late systole (mmHg) | 118 [113, 126] | 119 [113, 126] | 1.0 |
| WSS |  |  |  |
| Early systole (Pa) | 2.9 [2.7, 3.5] | 2.4 [1.7, 3.0] | 0.13 |
| Mid-systole (Pa) | 3.5 [3.0, 4.1] | 2.4 [1.7, 3.1] | 0.073 |
| Late systole (Pa) | 2.0 [1.2, 3.9] | 1.3 [1.0, 1.8] | 0.17 |
|  |  |  |  |
| Acute non-A non-B AD group (n=12) |  |  |  |
| Wall pressure |  |  |  |
| Early systole (mmHg) | 136 [131, 141] | 138 [134, 144] | 0.29 |
| Mid-systole (mmHg) | 159 [152, 168] | 162 [156, 170] | 0.41 |
| Late systole (mmHg) | 121 [113, 125] | 123 [115, 126] | 0.67 |
| WSS |  |  |  |
| Early systole (Pa) | 2.6 [1.8, 3.7] | 1.7 [1.1, 2.1] | 0.017 |
| Mid-systole (Pa) | 3.0 [1.9, 4.2] | 1.5 [0.92, 2.1] | 0.006 |
| Late systole (Pa) | 1.7 [0.84, 2.6] | 0.55 [0.23, 0.81] | 0.006 |

Data are shown as median [25^th^, 75^th^ percentile] values or number (%) of patients. TL: true lumen; FL: false lumen; AD: aortic dissection; WSS: wall shear stress; Pa: pascal.

|  | Tear width  <10mm  (n=9) | Tear width  >10mm  (n=10) | *P* value |
| --- | --- | --- | --- |
| Type of dissection |  |  |  |
| Acute type B AD | 5 (56%) | 2 (20%) | 0.17 |
| Acute non-A non-B AD | 4 (44%) | 8 (80%) | 0.17 |
| Age (years) | 51 [48, 53] | 62 [49, 68] | 0.24 |
| Male sex | 9 (100%) | 5 (50%) | 0.051 |
| Marfan syndrome | 0 (0%) | 2 (20%) | 0.47 |
| Hypertension | 8 (89%) | 7 (70%) | 0.58 |
| Uncomplicated course | 8 (89%) | 6 (60%) | 0.30 |
| Aortic rupture | 0 (0%) | 2 (20%) | 0.47 |
| Malperfusion | 1 (11%) | 3 (30%) | 0.58 |
| Cerebral | 0 (0%) | 1 (10%) | 1.0 |
| Kidney | 0 (0%) | 1 (10%) | 1.0 |
| Lower limb | 1 (11%) | 1 (10%) | 1.0 |
| Common orifice of the brachiocephalic trunk and the left common carotid artery | 3 (33%) | 1 (10%) | 0.30 |
| Distal extension of dissection |  |  |  |
| Limited to descending thoracic aorta | 0 (0%) | 1 (10%) | 1.0 |
| Abdominal aorta | 4 (44%) | 5 (50%) | 1.0 |
| Iliac artery | 5 (56%) | 4 (40%) | 0.65 |
| Entry location |  |  |  |
| Aortic arch | 3 (33%) | 6 (60%) | 0.37 |
| Descending thoracic aorta | 6 (67%) | 4 (40%) | 0.37 |
| Entry tear distance to LSA (mm) | 4.4 [0, 14.6] | 7 [3.5, 24.4] | 0.32 |
| TL/FL pressure ratio |  |  |  |
| at early systole | 1.0 [0.97, 1.03] | 1.01 [1.0, 1.03] | 0.28 |
| at mid-systole | 1.0 [0.96, 1.03] | 1.01 [1.0, 1.03] | 0.40 |
| at late systole | 1.0 [0.99, 1.02] | 1.0 [1.0, 1.01] | 0.72 |
| TL/FL pressure ratio >1.0 |  |  |  |
| at early systole | 5 (56%) | 9 (90%) | 0.14 |
| at mid-systole | 5 (56%) | 8 (80%) | 0.35 |
| at late systole | 6 (67%) | 7 (70%) | 1.0 |

**Table S3:** Patient/case characteristics and FL/TL pressure ratio, per size of the entry tear

Data are shown as median [25^th^, 75^th^ percentile] values or number (%) of patients are shown. AD: aortic dissection; LSA: left subclavian artery; TL: true lumen; FL: false lumen; WSS: wall shear stress; Pa: pascal.
